# Apple Sunburn: Physiological Mechanisms, Influencing Factors, and Advances in Sustainable Management Strategies

**DOI:** 10.3390/plants15213310

**Published:** 2026-10-26

**Authors:** Abdullah Hafeez, Yang Cao, Baisheng Ma, Quan Fang, Wenya Tian, Tuanhui Bai, Yifeng Feng

**Affiliations:** 1 College of Horticulture and Forestry, Tarim University/The National and Local Joint Engineering Laboratory of High Efficiency and Superior-Quality Cultivation and Fruit Deep Processing Technology of Characteristic Fruit Trees in Southern Xinjiang, Alar 843300, China; abdullahhafeez0306@gmail.com (A.H.); caoyang126129@163.com (Y.C.); mabaisheng16@163.com (B.M.); fangquan163@163.com (Q.F.); twy5412@163.com (W.T.); tuanhuibai@henau.edu.cn (T.B.); 2 Southern Xinjiang Distinctive Foresty & Pomology Technology Innovation Center, Xinjiang Production and Construction Corps, Alar 843300, China; 3 College of Horticulture, Henan Agricultural University, Zhengzhou 450046, China

**Keywords:** fruit quality, thermal stress, oxidative damage, evaporative cooling, reflective particle films, orchard management, climate resilience

## Abstract

Apple (*Malus domestica* Borkh.) is one of the most widely cultivated fruit crops globally. However, fruit sunburn, induced by excessive heat and intense solar radiation, poses a significant threat to apple production and fruit quality. Although numerous studies have investigated individual mitigation strategies, a comprehensive synthesis of sustainable management approaches and their practical applicability remains limited. This review provides an overview of advances in apple sunburn management, encompassing cultural practices such as canopy management and irrigation, physical protection methods including shade nets and evaporative cooling, protective treatments such as reflective sprays and particle films, and emerging approaches such as nanocoatings and gene editing. The review compares the reported effectiveness, physiological mechanisms, limitations, and practical considerations of these strategies. The evidence indicates that integrating complementary approaches may improve sunburn management, although their effectiveness varies with cultivar characteristics, climatic conditions, orchard practices, and resource availability. Accordingly, a practical framework is presented to guide strategy selection according to local sunburn risk, cultivar susceptibility, and economic feasibility. Future research priorities include improved risk-prediction models, standardized field trials, precision monitoring, and functional validation of candidate genes. This synthesis provides a basis for developing site-specific, economically feasible, and sustainable strategies to mitigate apple sunburn under changing climatic conditions.

## 1. Introduction

The global apple industry occupies a central position in international fruit production and trade, with market demand increasing over the past decade [1]. Despite its economic importance, apple production and fruit quality remain highly sensitive to environmental conditions [2]. Climate change is intensifying the risk of heat waves and episodes of excessive solar radiation in major apple growing regions, increasing the likelihood of sunburn damage during fruit development and maturation [3,4]. Consequently, apple sunburn is not only a fruit quality problem but also a growing challenge to orchard profitability, climate resilience, and the sustainability of apple production systems.

Apple sunburn is a physiological disorder caused by excessive solar radiation and elevated fruit-surface temperatures, resulting in visible peel injury and, in severe cases, substantial losses in marketable yield [5]. Three major forms are generally distinguished according to their symptoms and underlying physiological processes. Sunburn browning is characterized by brown or bronze discoloration associated with oxidative stress, membrane damage, and changes in phenolic metabolism. Sunburn necrosis represents more severe injury involving irreversible cell death and darkened, necrotic peel tissue. Photooxidative sunburn, also referred to as sunburn bleaching, develops when intense light exposure damages fruit that has insufficient photoprotective capacity, producing pale or bleached areas [3,6,7]. These forms represent related but physiologically distinct responses; their development depends on the interaction between radiation intensity, fruit-surface temperature, exposure duration, and the fruit’s capacity to acclimate to environmental stress. Therefore, a single temperature threshold should not be treated as universally predictive of sunburn across cultivars and growing conditions. Accurate differentiation among sunburn types is essential because injury severity, diagnostic criteria, and the effectiveness of mitigation strategies may differ.

Sunburn occurrence and severity are influenced by solar radiation, fruit-surface temperature, plant water status, and orchard management practices [8,9]. The effects of these factors are interdependent rather than independent. For example, high radiation increases the energy load on exposed fruit, while limited evaporative cooling under water-limited conditions may intensify thermal stress. Canopy architecture, fruit exposure, crop load, and the timing of fruit exposure also influence the local microclimate and the susceptibility of individual fruit [8,9,10]. In addition, cultivar characteristics affect peel pigmentation, photoprotective capacity, and market-quality requirements. Management decisions must therefore account for both sunburn susceptibility and potential trade-offs, particularly because interventions that reduce radiation exposure or fruit temperature can also alter red-color development, fruit quality, and orchard microclimate [7,9,11,12]. These interactions help explain why a treatment that performs well in one orchard may produce different results under another combination of climate, cultivar, and management conditions.

A range of sunburn mitigation strategies has been developed, including shade and photoselective nets, evaporative cooling, reflective particle films, and adjustments to orchard management [8,9,10,11,12]. Recent studies have also investigated integrated approaches that combine protective technologies with cultivar-specific orchard practices, while molecular studies have improved understanding of the biochemical and genetic responses associated with apple peel injury and tolerance [3,5,12,13,14]. Nevertheless, the effectiveness of these strategies cannot be evaluated solely by whether they reduce fruit temperature or visible sunburn. A meaningful comparison should consider quantitative changes in sunburn incidence and severity, fruit temperature, marketable yield, fruit coloration, water and energy requirements, implementation costs, and the persistence of protection under commercial growing conditions. These outcomes are not interchangeable: a reduction in fruit temperature does not necessarily translate into an equivalent reduction in sunburn incidence or an improvement in economic returns. Furthermore, the financial feasibility of nets, cooling systems, and repeated particle-film applications may differ considerably among production regions and orchard scales [9,10,12].

Despite progress in sunburn physiology and management, important gaps remain in translating experimental findings into reliable recommendations for commercial apple production. First, results obtained under controlled conditions or in individual orchards are not always directly comparable because studies differ in cultivar, rootstock, climate, treatment timing, sunburn classification, and assessment methods [7,13,15,16]. Second, comparative evidence on treatment effectiveness is often fragmented across studies, making it difficult to determine which interventions provide the greatest protection under particular environmental conditions and how their benefits compare with their costs. Third, cultivar-specific susceptibility and the trade-offs between sunburn prevention and red-color development remain important considerations when selecting management strategies [7,11,12,13]. Finally, although temperature measurements and risk-prediction models can support timely intervention, their practical value depends on reliable risk thresholds and their applicability to different cultivars, orchard systems, and climatic regions [15,16]. Addressing these limitations requires a critical synthesis that distinguishes demonstrated treatment effects from general recommendations, identifies inconsistencies in the available evidence, and evaluates the practical feasibility of alternative management approaches.

This review synthesizes current knowledge of apple sunburn, emphasizing the physiological mechanisms underlying injury-, environmental- and orchard-related factors influencing susceptibility, and conventional and emerging mitigation strategies. Beyond summarizing individual approaches, it critically compares their reported effectiveness, identifies sources of variation among studies, and examines the limitations of existing evidence across cultivars and production environments. Particular attention is given to cultivar-specific susceptibility, the tradeoffs between sunburn protection and fruit coloration, and the economic and environmental considerations associated with different interventions. The review also develops a practical framework for selecting management strategies according to climatic risk, cultivar susceptibility, water availability, orchard conditions, and economic constraints, while identifying research priorities for improving quantitative risk assessment and sustainable sunburn management. By linking physiological evidence with comparative treatment performance and practical decision making, this review aims to provide a stronger scientific basis for reducing sunburn losses and improving the resilience and profitability of apple production under a changing climate.

## 2. Physiological Mechanisms of Apple Sunburn Induced by Environmental Factors

**Figure 1:**
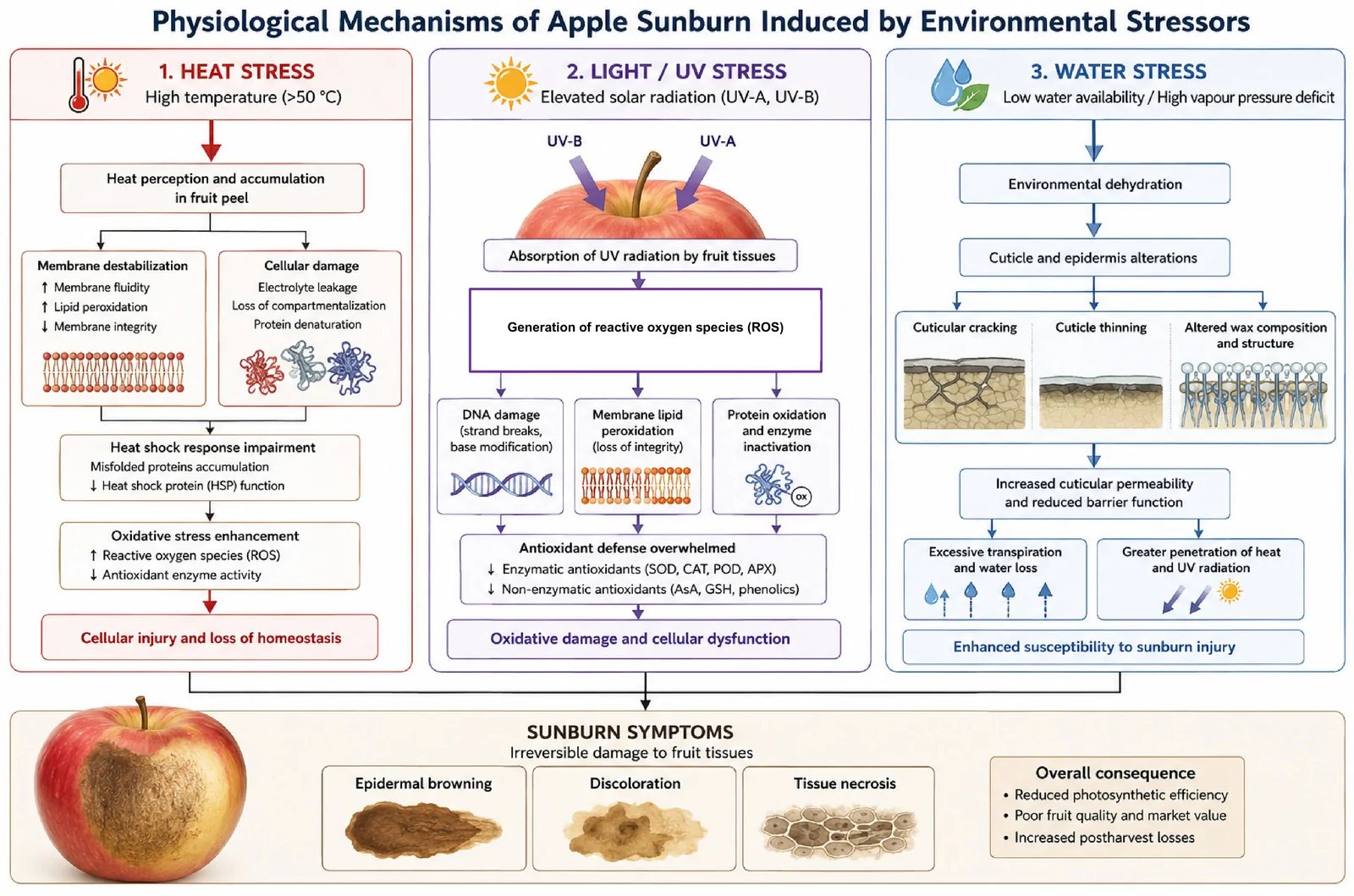
Excessive solar radiation, elevated fruit-surface temperature, and altered water status interact to affect apple peel physiology. Heat stress disrupts membrane integrity and protein homeostasis, while excessive light increases ROS production and may overwhelm photoprotective and antioxidant systems. Water imbalance and variation in cuticular properties modify fruit-surface protection. These interacting processes contribute to photooxidative bleaching, sunburn browning, and sunburn necrosis, depending on exposure intensity, duration, and cultivar susceptibility.

Apple sunburn is a physiological disorder caused by the interacting effects of excessive solar radiation, elevated fruit-surface temperature, and plant water status (Figure 1) [3]. Its development involves interconnected processes, including membrane damage, reactive oxygen species (ROS) accumulation, antioxidant defense, pigment metabolism, and changes in cuticular properties [17]. Understanding these mechanisms is essential for identifying factors underlying cultivar susceptibility and developing effective mitigation strategies [18]. The relative contribution of each process depends on stress intensity, exposure duration, fruit position, and cultivar characteristics.

### 2.1. Heat Stress Physiology

Heat stress is a major driver of apple sunburn, particularly when exposed fruit surfaces experience prolonged periods of elevated temperature. Severe thermal injury has been associated with fruit-surface temperatures of around 50–52 °C, whereas prolonged exposure to lower temperatures may also cause peel browning [19,20]. These values are indicative rather than universal thresholds because injury depends on temperature, exposure duration, and cultivar susceptibility. Fruit-surface temperature monitoring can therefore support sunburn risk assessment when combined with observations of injury severity [19].

Elevated temperatures can disrupt membrane integrity, increase membrane permeability, and promote lipid peroxidation, impairing cellular function in fruit-peel tissues [20,21]. Under severe stress, these processes may contribute to irreversible cellular injury and necrosis. Heat-shock proteins (HSPs) help maintain protein stability and cellular function under elevated temperatures. In apple, Che et al. [22] demonstrated that MdEF-Tu coordinates with MdHsp70 to enhance thermotolerance. Qiu et al. [17] further showed that MdHB7 enhances basal thermotolerance through ROS scavenging and lignin accumulation. These findings identify molecular and biochemical components of apple heat tolerance, although their direct contributions to reduced sunburn incidence require further validation under orchard conditions. Beyond membrane-associated damage, high-temperature stress also induces dynamic changes in stress granule formation and regulation, representing an additional mechanism through which plant cells respond to thermal stress [21].

### 2.2. Light- and UV-Induced Damage

Excessive solar radiation contributes to apple sunburn through photochemical stress and ROS accumulation, particularly when high irradiance coincides with elevated fruit-surface temperatures [23,24]. Heat stress primarily affects membrane integrity and protein function, whereas excessive light can overwhelm photoprotective mechanisms and intensify oxidative stress. These pathways overlap under field conditions, complicating the separation of thermal and radiation-induced injury.

Apple peel contains flavonoids, anthocyanins, and other phenolic compounds that contribute to UV screening and antioxidant defense [25,26,27]. Solovchenko and Schmitz-Eiberger [27] demonstrated the importance of skin flavonoids in UV-B protection, while Merzlyak et al. [26] examined the contribution of apple pigments to photoprotection. However, protective capacity depends on pigment composition, distribution, and environmental exposure; consequently, pigment abundance alone cannot reliably predict sunburn resistance.

When radiation exposure exceeds the capacity of protective systems, oxidative stress can contribute to pigment alteration and cellular injury [28,29,30]. Torres et al. [28] demonstrated that photooxidative stress activates the phenylpropanoid pathway, ethylene-related processes, and lignin accumulation in apple fruit. Photooxidative bleaching, sunburn browning, and sunburn necrosis should therefore be distinguished when assessing physiological responses and treatment effectiveness. ROS accumulation and antioxidant activity can help characterize stress responses, but these indicators should be interpreted alongside fruit-surface temperature and standardized assessments of visible injury.

### 2.3. Water Stress and Cuticular Regulation

Water status influences apple sunburn susceptibility by affecting tree water relations and responses to high evaporative demand. The fruit cuticle and associated waxes limit nonstomatal water loss and protect underlying tissues. Soil-water availability, root water uptake, canopy transpiration, and fruit exposure jointly influence water balance under hot conditions [23,31,32,33]. Severino et al. [23] examined relationships between sunburn damage and stem and fruit water potential in ‘Brasil Gala’, ‘Cripps Pink’, and ‘Granny Smith’ apples, highlighting the relevance of water relations to cultivar-specific responses.

Cuticular integrity and wax composition influence fruit-surface properties and barrier function [34,35,36]. Jiang et al. [33] demonstrated that MdBT2 regulates nitrogen-mediated cuticular wax biosynthesis through an MdMYB106–MdCER2L1 signaling pathway. This finding provides a molecular basis for investigating cuticle development, although the direct relationship between wax characteristics and sunburn resistance requires validation using measurements of cuticle properties and injury incidence.

Abscisic acid (ABA) contributes to water-deficit signaling and stomatal regulation [37,38]. Stomatal closure can conserve water but may also reduce evaporative cooling, making its net effect on fruit thermal stress dependent on environmental conditions. Irrigation management is therefore important for maintaining tree water status, but additional water alone cannot be assumed to prevent sunburn.

Ethylene-related processes also participate in apple fruit responses to photooxidative stress. Torres et al. [28] identified ethylene-associated responses alongside phenylpropanoid metabolism and lignin accumulation. However, the specific contribution of ethylene signaling to sunburn tolerance remains insufficiently characterized and requires further investigation.

Overall, heat stress, excessive radiation, water relations, and cuticular properties interact to determine apple sunburn susceptibility. Their relative importance varies with cultivar and orchard conditions, supporting integrated assessments of fruit-surface temperature, water status, peel physiology, and standardized injury severity for improved risk prediction and management.

## 3. Factors Influencing the Occurrence and Severity of Apple Sunburn

**Figure 2:**
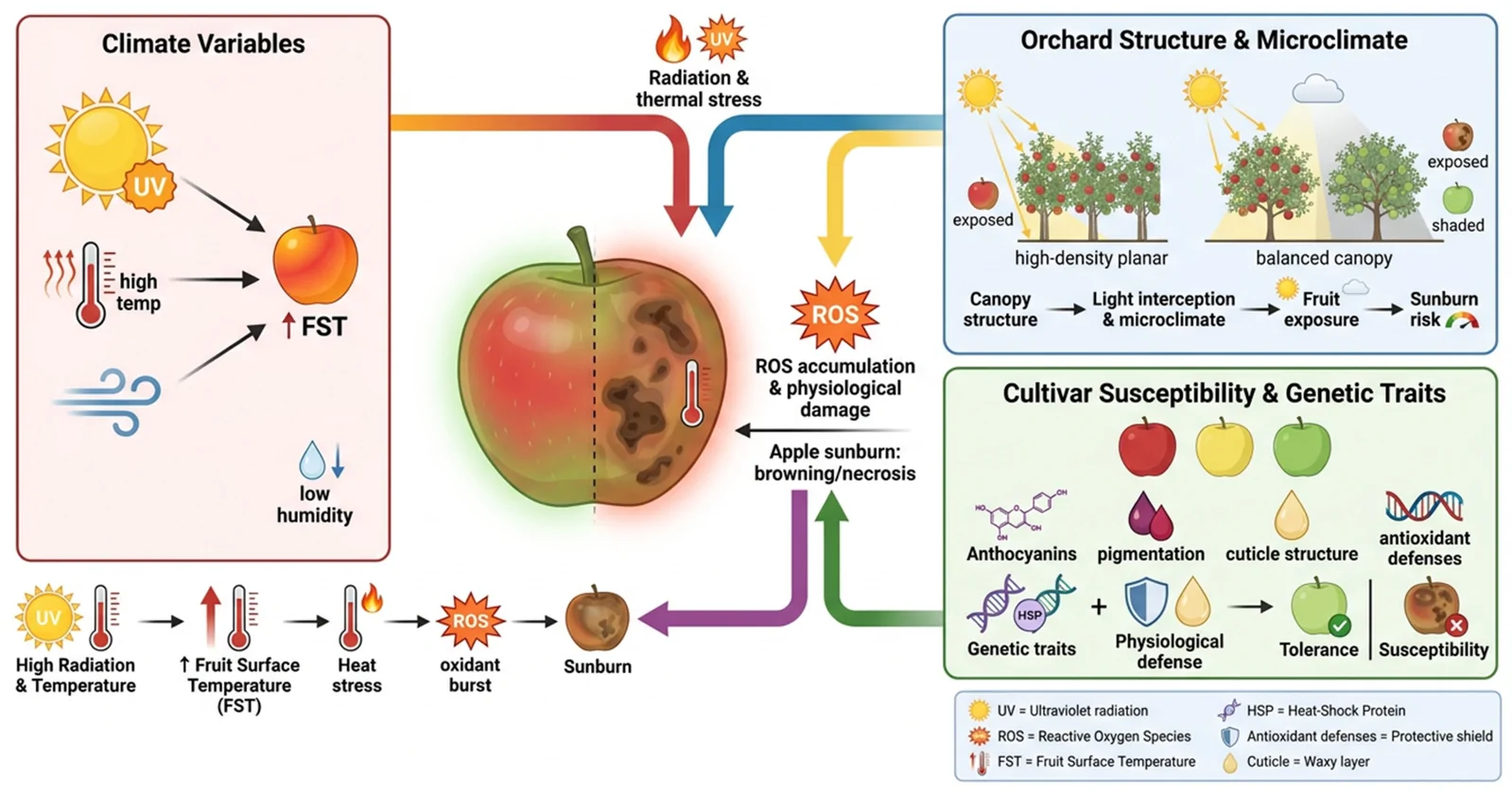
Climatic, orchard, and cultivar-related factors influencing the occurrence and severity of apple sunburn. Solar radiation, air temperature, relative humidity, and wind influence FST and radiation exposure, while canopy architecture and fruit position modify the orchard microclimate. Cultivar-dependent traits, including peel pigmentation, cuticle characteristics, antioxidant defenses, and genetic stress responses, contribute to variation in physiological tolerance. Excessive radiation and thermal stress can promote ROS accumulation and physiological damage, contributing to sunburn browning and necrosis. Sunburn risk reflects the interaction between environmental exposure and fruit susceptibility.

The occurrence and severity of apple sunburn are influenced by climatic conditions, orchard structure, and cultivar susceptibility. These factors interact to determine fruit-surface temperature, radiation exposure, and physiological tolerance, resulting in substantial variation in sunburn risk among orchards and production regions. Understanding these interactions is essential for developing effective and site-specific management strategies. The interactions among climatic variables, orchard structure, and cultivar-related traits influencing apple sunburn are summarized in Figure 2.

### 3.1. Climate Variables

The primary climatic factors influencing apple sunburn include solar radiation, air temperature, relative humidity, wind velocity, and heatwaves. High solar radiation and elevated air temperatures increase fruit-surface temperature (FST), potentially exceeding the thermal tolerance of exposed fruit [19]. Relative humidity and wind velocity modify the fruit energy balance: low humidity can increase evaporative demand, whereas wind can promote convective heat dissipation, although its cooling effect depends on wind speed, radiation, and ambient conditions [39,40]. Prolonged heatwaves are particularly damaging because sustained thermal exposure increases the risk of browning and necrosis [18]. Consequently, air temperature alone may be insufficient for predicting injury, and direct FST measurements provide a more relevant indicator of fruit-level thermal risk [19,40].

Climate change is expected to increase heat-related challenges in many fruit-growing regions, strengthening the need for climate-adaptive orchard management [41,42,43]. Protective netting and emerging approaches, including dynamic agrivoltaic systems, offer potential means of modifying radiation exposure and fruit temperature [41,42]. However, their effectiveness depends on local climatic conditions, system design, and implementation costs. Management decisions should therefore consider both the severity of local heat risk and the economic feasibility of available protection methods.

### 3.2. Orchard Structure and Microclimate Regulation

Orchard architecture influences sunburn susceptibility by determining canopy shading, fruit exposure, and local microclimatic conditions. High-density systems with narrow, planar canopies may expose more fruit to direct solar radiation than canopies providing greater natural shade, although the actual risk depends on canopy orientation, pruning, and fruit distribution [5,43,44]. Fruit position within the canopy is particularly important because exposed fruit generally experiences greater radiation and thermal loads than shaded fruit. Racskó et al. [44] demonstrated that sunburn incidence is influenced by rootstock and fruit position within the canopy, emphasizing that orchard structure and rootstock selection can jointly affect injury risk.

Row orientation, slope, and exposure to afternoon sunlight can further modify fruit temperature. Western and other strongly sun-exposed canopy positions may experience high thermal loads during hot afternoons, but the magnitude of this effect depends on latitude, terrain, season, and orchard layout [19,40]. Elevation may also modify temperature and UV exposure; however, its net effect on sunburn cannot be inferred from elevation alone as radiation, atmospheric conditions, and local climate interact.

Canopy management must balance fruit exposure with light interception and coloration. Aggressive summer pruning or leaf removal can abruptly expose previously shaded fruit to intense radiation, increasing the risk of sunburn, particularly when performed during hot weather [19,43]. Gradual canopy management that maintains adequate leaf cover around susceptible fruit may reduce excessive exposure while preserving canopy productivity. Protective netting and other microclimate-modifying approaches should be selected according to orchard architecture, local heat risk, and their effects on fruit coloration and yield [41,43,45].

### 3.3. Cultivar Susceptibility and Genetic Traits

Genetic variation contributes substantially to differences in apple sunburn susceptibility. Cultivars differ in peel pigmentation, antioxidant composition, surface characteristics, and responses to thermal and radiation stress [2,46,47]. These traits may influence photoprotection and the severity of visible injury, but no single characteristic reliably predicts resistance across all environmental conditions. For example, Ross et al. [46] investigated changes in fruit-skin absorbance and heat-stress response compounds in relation to sunburn-browning severity in ‘Buckeye Gala’, providing evidence for associations between peel optical properties, stress responses, and injury severity.

Peel coloration should not be treated as a universal indicator of sunburn resistance. Anthocyanins and other phenolic compounds can contribute to photoprotection, while differences in surface optical properties may influence radiation absorption and fruit heating [26,27,46]. Shade netting and reflective mulches have also been evaluated in ‘Gala Baigent’ and ‘Fuji Raku Raku’, demonstrating the importance of considering cultivar and management method together when assessing fruit quality and yield [47]. These findings support cultivar-specific evaluation rather than assuming that a treatment effective for one cultivar will provide equivalent protection in another.

Genetic studies offer additional opportunities to understand stress tolerance. Yao et al. [48] characterized the apple HSP20 gene family and identified potential members associated with temperature-stress responses. Such molecular findings provide candidate targets for investigating thermotolerance, but their contribution to field-level sunburn resistance requires direct phenotypic validation. Integrating cultivar screening, physiological measurements, and genetic analyses may therefore improve the identification of suitable cultivars and support the development of targeted sunburn management strategies.

## 4. Traditional Sunburn Control Methods in Apple Orchards

Traditional apple sunburn management relies on cultural practices, evaporative cooling, and protective netting. These approaches primarily reduce fruit exposure or thermal stress, but their effectiveness depends on climatic conditions, water availability, orchard design, and cultivar. Their performance should therefore be evaluated in terms of sunburn reduction, fruit quality, resource requirements, and economic feasibility.

### 4.1. Cultural Practices: Irrigation and Canopy Management

Cultural practices play a key role in sunburn management by improving orchard conditions and minimizing fruit exposure to environmental stressors. Effective irrigation is essential for maintaining tree water status and supporting fruit development. During hot periods with limited rainfall, adequate water supply helps maintain normal physiological processes and reduces the additional stress associated with water deficits [35,37]. However, irrigation alone may not prevent sunburn when fruit surfaces are exposed to excessive solar radiation and high temperatures.

Canopy management is another important strategy for reducing sunburn. Adjusting canopy structure helps regulate the amount of direct sunlight reaching fruit and moderates fruit temperature [5,36]. Appropriate pruning should maintain sufficient leaf cover while allowing adequate light penetration for fruit development and coloration. Excessive pruning or sudden leaf removal during hot periods may expose previously shaded fruit to intense radiation, increasing sunburn risk.

Tree training systems also influence fruit position and exposure to sunlight. Fruit growth and quality vary with canopy position, reflecting differences in the microenvironment experienced by individual fruit [43]. High-density and planar training systems may increase the exposure of fruit positioned along the outer canopy, whereas greater self-shading can reduce direct radiation but may also limit light penetration. Therefore, canopy architecture should be managed according to orchard design, cultivar characteristics, and local climatic conditions. Combining appropriate irrigation with canopy management can help maintain tree water status while limiting fruit exposure; however, the effectiveness of this combination depends on the severity of heat stress and the characteristics of the production system [36,37].

### 4.2. Evaporative Cooling: Principle and Application

Evaporative cooling is widely used to manage apple sunburn by reducing fruit-surface temperature through water evaporation. Overhead sprinklers and micro-sprayers distribute water over the canopy and fruit surfaces, where evaporation dissipates heat and alleviates thermal stress [49,50,51,52,53,54]. The cooling effect depends on relative humidity, wind speed, air temperature, and evaporation rate. Evaporation generally proceeds more rapidly under low-humidity conditions, whereas high relative humidity limits cooling efficiency [40,54].

Evaporative cooling is particularly useful during periods of extreme heat, when fruit-surface temperature monitoring can help determine the timing of application [12,19]. However, its effectiveness depends on the availability of sufficient water and suitable atmospheric conditions. High water demand can increase operating costs and restrict its use in water-scarce regions. Prolonged wetting of fruit and foliage may also increase disease-management challenges under favorable conditions for pathogen development.

Weather-informed scheduling and fruit-surface temperature monitoring can improve application timing and reduce unnecessary water use [12,19]. Nevertheless, the economic and environmental feasibility of evaporative cooling depends on water availability, energy requirements, infrastructure, and the expected reduction in sunburn-related losses. It is therefore most suitable where reliable water supplies and favorable evaporative conditions allow effective cooling at an acceptable cost.

### 4.3. Shade Nets: Types, Efficacy, and Key Considerations

Shade nets reduce incoming solar radiation, moderate orchard microclimate, and help protect fruit from excessive heating [24,36,55,56,57]. Their effectiveness depends on net material, color, mesh structure, shading intensity, climatic conditions, and cultivar susceptibility. The choice of shading intensity should balance sunburn prevention against the need to maintain sufficient light for photosynthesis and fruit coloration. Higher shading levels may be beneficial under intense radiation, whereas excessive shading can reduce light availability and affect fruit quality.

Studies have demonstrated that netting responses vary among apple cultivars. Olivares-Soto et al. [56] reported differential effects of net-based sunburn control on the antioxidant properties of ‘Gala’ and ‘Fuji’ apple peels, while photoselective netting has also been evaluated for its effects on ‘Honeycrisp’ fruit quality [24]. These findings indicate that netting should be assessed in relation to both sunburn incidence and market quality characteristics. Protective nets may also reduce orchard water requirements under certain conditions [58], although the magnitude of these benefits depends on the production environment and net configuration.

A major limitation of conventional fixed netting is its potential effect on red fruit coloration. Reduced light availability may restrict pigment development, particularly in red-skinned cultivars [50,59]. Retractable netting offers greater flexibility by allowing protection during high-risk heat periods while permitting greater light exposure when conditions are suitable for fruit coloration. Research on ‘Honeycrisp’ has examined retractable netting and evaporative cooling for sunburn control and red color development [12], while the timing of net retraction before harvest has also been investigated [52]. Reflective groundcovers may further improve fruit skin color under protective netting [59].

The comparative performance of individual sunburn mitigation interventions is summarized in Table 1, which brings together reported effects on fruit-surface temperature, sunburn incidence, fruit quality, and relevant technical characteristics. The evidence demonstrates that treatment responses can differ among cultivars and experimental conditions. For example, netting may reduce sunburn risk while also modifying red coloration, whereas some protective approaches affect water use or fruit temperature without providing directly comparable measurements of sunburn incidence. These differences emphasize the importance of evaluating multiple outcomes rather than ranking interventions on the basis of a single parameter. Table 1 therefore provides intervention-level evidence that informs the broader comparison of sustainable management strategies presented in Table 2.

## 5. Innovative and Advanced Technologies for Apple Sunburn Control

With the increasing severity of apple sunburn associated with changing climatic conditions, innovative mitigation technologies have been developed to reduce fruit damage and maintain market quality. These approaches include protective particle films, sunburn-reducing sprays, advanced cooling and covering systems, biostimulants, and emerging genetic strategies. Their effectiveness varies with application method, environmental conditions, cultivar susceptibility, and production costs; therefore, their practical value should be assessed alongside their effects on fruit quality and orchard sustainability [5,7,65].

### 5.1. Protective Particle Films and Nanocoatings

Protective particle films, including kaolin, calcium carbonate, talc, and silica-based formulations, have attracted attention as potential tools for mitigating apple sunburn [7,66]. These materials form a reflective layer on fruit surfaces, scattering or reflecting part of the incoming solar radiation and potentially reducing fruit-surface temperature. Their protective effects depend on formulation, application timing, and uniform surface coverage [4,5]. Calcium carbonate-based foliar sprays have also been investigated for protecting fruit quality during extreme heat events [4].

Despite their potential benefits, particle films have several practical limitations. Residues may accumulate on fruit surfaces, affecting visual appearance and potentially reducing marketability if removal is difficult before packing [5]. Repeated applications may be necessary to maintain adequate coverage as fruits expand or deposits are lost through rainfall and orchard operations. Consequently, application concentration and timing should balance protective performance with residue accumulation, fruit appearance, and application costs. Improving adhesion and persistence while maintaining acceptable fruit-surface appearance remains an important consideration in the development of these formulations.

Nanomaterial-based coatings represent a potential extension of particle-film technology, but their effectiveness and safety require further validation under commercial orchard conditions. Although some nanomaterials possess optical properties that may be useful for modifying radiation exposure, their performance depends on particle composition, concentration, formulation, and interaction with plant tissues. In particular, zinc oxide and titanium dioxide nanoparticles may exhibit photocatalytic activity under suitable conditions, potentially generating reactive oxygen species. Their use therefore requires careful evaluation of dose-dependent phytotoxicity, environmental fate, residue behavior, and regulatory acceptability before they can be considered practical alternatives to conventional particle films. Comparative field studies are needed to establish whether these materials provide consistent advantages in sunburn prevention, fruit quality, persistence, and cost.

### 5.2. Sunburn-Reducing Sprays and Plant Growth Regulators

Sunburn-reducing sprays provide an alternative approach to apple sunburn management and include antitranspirants, antioxidants, and UV-absorbing compounds [67]. Antitranspirants form a protective film on plant surfaces that reduces water loss, although their effectiveness in preventing sunburn depends on formulation and application conditions [68]. Antioxidants, such as ascorbic acid, may help alleviate oxidative stress by scavenging reactive oxygen species (ROS) generated under excessive radiation [69,70]. UV-absorbing compounds, including certain flavonoids and other natural products, may also reduce radiation penetration and associated oxidative injury [4]. The effectiveness of these treatments depends on their composition, persistence, application timing, and frequency. Repeated applications may be necessary to maintain protection throughout the growing season, particularly when residues are lost through rainfall or fruit expansion. Naturally derived compounds, such as rutin and quercetin, are potential candidates for further investigation; however, their effectiveness and suitability for commercial or organic apple production require validation under field conditions.

Plant growth regulators (PGRs) represent another potential approach to improving apple responses to environmental stress [70]. These compounds regulate physiological processes involved in stress responses and fruit development. Their contribution to sunburn mitigation depends on the specific regulator, application concentration, and developmental stage of the fruit. Although PGRs may complement physical protection and cooling strategies, their effectiveness should be evaluated directly in terms of sunburn incidence and fruit quality. Excessive or poorly timed applications may adversely affect fruit growth and maturation. Abscisic acid (ABA), for example, participates in stress-response signaling and may influence fruit tolerance to environmental stress [35]. However, its practical application for sunburn control requires further assessment to establish effective application timing and avoid undesirable effects on fruit ripening.

### 5.3. Artificial Shade Structures and Smart Covering Systems

The adoption of artificial shade structures and smart covering systems for apple sunburn management is increasing. These systems include reflective orchard groundcovers, photoselective nets, and retractable netting structures [50,71]. By modifying light distribution and orchard microclimate, these systems can help reduce sunburn risk while maintaining fruit quality. Their effectiveness depends on climatic conditions, cultivar susceptibility, and the design and timing of deployment.

Reflective orchard films, deployed as groundcovers between tree rows rather than as overhead shade, are used to capture and redirect light into the lower canopy [59,72]. These films reflect incoming sunlight upward, increasing light availability to inner canopy fruit and potentially enhancing red skin coloration [59,72]. However, because reflective groundcovers increase light exposure rather than directly shading fruit, their effects on sunburn risk should be evaluated alongside local radiation intensity and fruit-surface temperature.

Cost–benefit analyses of these systems vary depending on orchard size and geographical location. The installation costs of retractable netting and reflective groundcovers can be substantial, warranting careful evaluation of their economic feasibility [60]. However, improvements in marketable fruit quality and reductions in sunburn-related losses may help offset the initial investment, depending on local production conditions [60]. For small orchards, installation and maintenance costs may be prohibitive, highlighting the need for more affordable alternatives. Solar powered retractable systems may reduce dependence on conventional energy sources; however, their long-term energy savings, environmental benefits, and economic performance require further evaluation under commercial orchard conditions.

### 5.4. Preharvest Application of Biostimulants

Biostimulants, including seaweed extracts, amino acids, and humic substances, have demonstrated potential for improving plant growth, fruit quality, and tolerance to abiotic stress [73,74]. Seaweed extracts contain biologically active compounds, including phytohormones, which can promote plant growth and influence stress responses [75]. However, their direct effectiveness in reducing apple sunburn requires further evaluation under field conditions. Certain amino acids, such as proline and glutamine, are involved in plant stress responses through osmotic adjustment and the stabilization of cellular structures [70,73,76,77]. Humic substances can also influence nutrient uptake and plant physiological processes, thereby supporting overall tree and fruit development [76].

These biostimulants may contribute to stress tolerance through complementary physiological mechanisms, although their effects depend on product composition, application timing, concentration, and environmental conditions. Their application may influence antioxidant defense and stress-associated responses; however, the induction of heat-shock proteins or antioxidant activity should not be interpreted as direct evidence of reduced sunburn incidence without supporting experimental data [61,75,76]. Compared with some conventional chemical treatments, biostimulants may offer an alternative for sustainable orchard management. Nevertheless, their practical value as sunburn-control treatments should be established through field trials assessing sunburn severity, fruit quality, yield, and application costs.

### 5.5. Genetic Engineering and Gene Editing Approaches

Genetic engineering holds considerable potential for enhancing resistance to apple sunburn. A key strategy involves the overexpression of genes associated with antioxidant pathways. Antioxidants are critical for neutralizing reactive oxygen species (ROS) generated during UV and heat stress. By modulating the expression of genes involved in antioxidant defense, including those encoding superoxide dismutase (SOD), catalase, and peroxidase, researchers may strengthen the fruit’s capacity to withstand oxidative stress and reduce sunburn-related cellular damage [78].

In addition to antioxidant-based strategies, CRISPR-based gene editing has emerged as a promising tool for investigating and potentially improving sunburn resistance. This technology enables targeted modifications of the apple genome, allowing researchers to investigate genes involved in stress-response pathways [78]. For example, genes associated with heat-stress responses, cuticle formation, and photoprotection may provide candidate targets for future research [79,80]. The heat-shock proteins MdHSP70 and MdHSP90 are involved in heat-stress responses [22], while MdCER1 has been investigated in relation to wax biosynthesis and drought stress [79]. Similarly, research on MdSCL8 and FLS1 has examined the regulation of flavanol accumulation in apple [80]. These genes provide potential targets for functional investigation; however, their direct contributions to apple sunburn resistance remain to be established. Genetic engineering and gene editing may therefore provide valuable tools for elucidating the molecular mechanisms underlying sunburn tolerance. Further functional validation and field evaluation are required to determine whether modifications to these pathways can produce apple cultivars with consistently reduced sunburn damage without compromising fruit quality or other desirable traits.

### 5.6. Integrated Sunburn Management (ISM) Approaches

In recent years, there has been a growing emphasis on integrating diverse management strategies, including cultural, chemical, and structural approaches, to combat sunburn in apple orchards. ISM combines multiple protective measures to maintain fruit quality while minimizing sunburn damage. These strategies combine complementary interventions to reduce fruit exposure and improve stress management. Effective apple orchard management depends on climate adapted orchard design and the cost effectiveness of sunburn control methods. Integrated approaches may provide more consistent protection than individual methods; however, their effectiveness depends on the combination of treatments and local growing conditions [7,12,60].

Climate-smart orchards are a cornerstone of ISM, helping growers mitigate sunburn damage under increasingly variable climatic conditions. Such designs incorporate high-density planting, reflective groundcovers, and optimized row orientation to balance light interception and sunburn risk. While high-density planting improves land use efficiency, modern intensive, planar canopy architectures, such as two-dimensional (2D) spindles or multi-leader fruiting walls, can reduce vegetative self-shading. Consequently, a greater proportion of fruit may be exposed to direct afternoon radiation, increasing sunburn risk unless appropriate protective measures, such as netting or evaporative cooling, are implemented [7,60]. Reflective ground fabrics or mulches represent another component of climate-smart orchard management. Rather than directly shading trees, these materials reflect sunlight upward into the lower and inner canopy, increasing light availability and potentially enhancing anthocyanin accumulation and red skin coloration [59,71]. However, increased radiation exposure may also increase the thermal load on fruit under high-temperature conditions. Organic or biodegradable mulches may additionally help conserve soil moisture and alleviate water stress during periods of extreme heat, depending on their properties and application [59].

Optimized row orientation may also influence solar radiation distribution and fruit exposure within the canopy. The suitability of a particular orientation depends on latitude, local solar trajectories, orchard architecture, and prevailing climatic conditions. North–south (N–S) rows can expose fruit on the western side of the canopy to intense afternoon sunlight, when high ambient temperatures may further increase sunburn risk [12]. Alternative orientations, including northwest–southeast (NW–SE), may modify the timing and distribution of solar exposure under particular growing conditions [7,60,81]. However, no single row orientation can be considered universally optimal for sunburn prevention; its effectiveness should be assessed in relation to local radiation patterns, canopy structure, and the need to maintain adequate light for fruit development and coloration. The selection of appropriate sunburn mitigation strategies should consider the interaction between climatic risk, cultivar susceptibility, orchard design, resource availability, and economic feasibility. Although individual approaches can effectively reduce sunburn under specific conditions, their performance varies among production regions and management systems. A quantitative comparison of major sustainable sunburn mitigation strategies, including their mechanisms, reported effectiveness, limitations, and practical considerations, is presented in Table 2. This synthesis highlights the complementary roles of different approaches: physical methods, such as protective netting and evaporative cooling, can provide direct protection against excessive solar radiation and fruit heating, whereas reflective materials, physiological treatments, and genetic approaches offer additional options for integrated, long-term sunburn management. Together with the intervention-level evidence summarized in Table 1, this comparison supports the selection of strategies according to orchard conditions, resource availability, and management objectives.

### 5.7. Practical Framework for Selecting Apple Sunburn Management Strategies

The selection of apple sunburn management strategies should consider climatic risk, cultivar susceptibility, orchard conditions, resource availability, and cost. Strategies can be grouped into four complementary levels: foundational practices (irrigation and canopy management), direct protection (netting and evaporative cooling), supplementary treatments (particle films and biostimulants), and long-term approaches (cultivar selection and genetic improvement). This hierarchy reflects their functions and implementation timescales rather than a universal ranking of effectiveness.

In orchards exposed to extreme heat, growers should prioritize early risk assessment, fruit-surface temperature monitoring, and suitable protective measures. In water-limited regions, water and energy requirements should guide intervention choices, while cultivars requiring red coloration need careful consideration of shading intensity and timing. Where investment is limited, irrigation scheduling, canopy management, and fruit-exposure control should be evaluated first. Emerging treatments should remain supplementary until they can be adequately validated under field conditions. Management effectiveness should be assessed using sunburn severity, fruit coloration, marketable yield, resource use, and cost to support site-specific decisions.

## 6. Conclusions and Future Perspectives

In conclusion, considerable progress has been made in managing apple sunburn through integrated approaches combining orchard management, physical protection, biochemical treatments, and genetic improvement. The effectiveness of these strategies varies with climatic conditions, cultivar susceptibility, orchard design, and resource availability. Physical interventions, including evaporative cooling and protective netting, provide an important means of reducing thermal stress and excessive radiation exposure, whereas particle films and other biochemical treatments may offer complementary protection [4,12,24,54,56,60,61]. However, no single method is universally optimal, and treatment selection should consider both sunburn reduction and its effects on fruit quality, marketable yield, and production costs. Evaporative cooling may be suitable where water supplies are reliable and evaporative conditions are favorable, whereas protective netting may be preferable in orchards experiencing recurrent sunburn. Biochemical treatments can complement these approaches where their effectiveness has been demonstrated under relevant field conditions.

**Figure 3:**
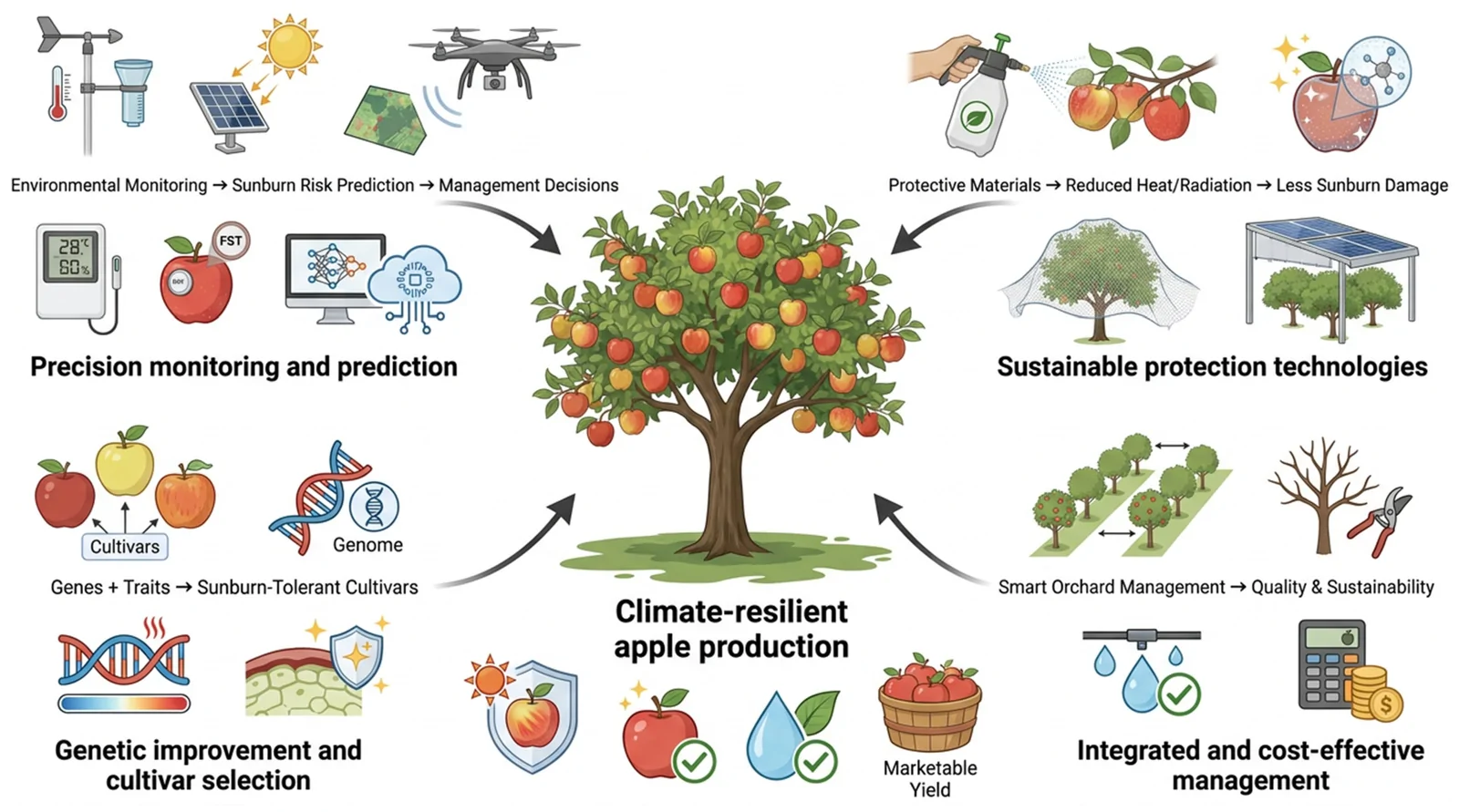
Integrated management approaches and future research priorities for mitigating apple sunburn. Orchard management, physical protection, biochemical treatments, and genetic improvement contribute to sunburn mitigation, with their suitability determined by local climate, cultivar susceptibility, orchard characteristics, and resource availability. Future priorities include predictive models incorporating FST and microclimate data, improved particle films and nanocoatings, precision interventions supported by AI and remote sensing, standardized comparative field trials, and functional validation of candidate genes. Integrating these approaches with assessments of fruit quality, marketable yield, resource use, and economic feasibility can support sustainable, climate resilient apple production.

Economic feasibility is equally important for translating research findings into practical orchard management. Although advanced cooling systems and retractable netting may provide effective protection, their adoption depends on installation and operating costs, water and energy requirements, orchard size, and the value of marketable fruit. Growers should therefore prioritize preventive measures according to local sunburn risk, cultivar susceptibility, available resources, and expected economic returns, combining methods when their benefits justify the additional costs. Region-specific recommendations are particularly important because the effectiveness of a treatment under one climatic condition may not be directly transferable to another. The integrated management approaches and future research priorities for mitigating apple sunburn are summarized in Figure 3.

Future research should prioritize the development of region- and cultivar-specific predictive models that integrate fruit-surface temperature, solar radiation, humidity, and orchard characteristics to improve sunburn risk assessment. Further investigation is needed to improve the field persistence, safety, and economic feasibility of environmentally compatible particle films and nanocoatings [82]. Artificial intelligence (AI), remote sensing, and real-time microclimate monitoring could support the precise timing of cooling and shading interventions while reducing unnecessary resource use. Standardized comparative field trials should quantify sunburn incidence and severity, marketable yield, fruit quality, water and energy consumption, and treatment costs across cultivars and production regions. In parallel, functional validation of candidate genes associated with heat responses, cuticle properties, and photoprotection is needed to establish their direct contributions to sunburn resistance. Ultimately, integrating validated technologies with cultivar-specific recommendations and cost–benefit assessments will provide a stronger basis for sustainable, climate-resilient apple production.

## Figures and Tables

**Table 1:** Quantitative Synthesis and Comparative Efficacy of Sunburn Mitigation Strategies.

Mitigation Strategy	Technical Matrix & Mechanism	FST/Sunburn Efficacy	Fruit Quality & Physiology	References
Photoselective nets—‘Honeycrisp’	Pearl, blue and red HDPE nets; 19–23% shading. Reduced PAR by 22–25% and UV by ~33%; altered spectral quality.	FST not measured. Sunburn-free fruit increased to 94–97% with blue and 88–92% with red nets vs. 52–71% in uncovered control.	Reduced red overcolor; red netting had lowest bitter-pit incidence; firmness largely unaffected.	[24]
Red/pearl nets—‘Gala’ and ‘Fuji’	20% shading; reduced IR by 8%, PAR by 21–25% and UV by 25–29%.	FST ↓ ~5 °C. Combined moderate + severe sunburn decreased 15% in ‘Gala’ and 4% in ‘Fuji’.	Red net increased anthocyanins/FRAP in ‘Gala’; pearl net reduced antioxidant compounds in ‘Fuji’.	[56]
Fixed white net—‘Rosy Glow’	Reduced solar radiation ~12%, wind > 36% and reference ET ~12%.	FST and sunburn not measured.	Transpiration ↓11%; irrigation requirement ↓4%; water productivity increased.	[58]
Conventional/photoselective nets—‘Gala Redlum’	Black, grey, white, red and yellow HDPE nets (9.6–22.4% shading); grey net provided strongest thermal moderation.	Grey net reduced fruit temperature by 9.1 °C vs. control at 14:00 in 2024. Sunburn loss: 5.64% control vs. 0.51% grey and 0.69% black.	Photosynthetic performance generally improved under nets; red/yellow nets altered fruit coloration.	[60]
Retractable net + evaporative cooling—‘Honeycrisp’	20% white net; evaporative cooling activated at 30 °C.	Netting: FST ↓2.5 ± 0.2 °C. Cooling: FST ↓2.7 ± 0.9 °C. Severe sunburn: ~7% control vs. 2.3% retained net and 3.2% with cooling.	Net retraction improved red color without increasing severe sunburn; cooling increased fruit weight in 2021.	[50]
Evaporative cooling	Overhead irrigation removes heat through evaporation; temperature-based pulsing can optimize water use.	Target fruit-core temperature: 30–32 °C; no single FST/sunburn reduction reported.	Primarily supports thermal management and water-use optimization.	[54]
Calcium carbonate foliar protectant—‘Fuji’	Reflective calcium carbonate coating reduces absorbed solar radiation.	Sunburn: 79% → 70%; severe sunburn: 37% → 28%. FST not measured.	Fruit asymmetry ↓17 percentage points; no significant effect on major quality or leaf gas-exchange traits.	[4]
Comparative mitigation—‘Honeycrisp’	20% netting, evaporative cooling, Raynox Plus^®^ and ScreenDuo^®^.	Netting produced the strongest response; sunburn incidence reduced >50% vs. control in the significant 2016 experiment. FST generally decreased under treatments.	Yield and fruit weight largely unaffected; netting tended to reduce red coloration.	[61]

Noted: ↓ represented for decreasing; → represented for ‘from’ and ‘to’.

**Table 2:** Quantitative comparison of sustainable management strategies for reducing apple sunburn.

Management Strategy	Mechanism of Action	Quantitative Effectiveness	Limitations and Economic Considerations	References
Protective netting and retractable netting systems	Reduces incoming solar radiation, modifies orchard microclimate, and lowers fruit-surface temperature while maintaining fruit development conditions	Retractable netting combined with evaporative cooling reduced sunburn incidence in ‘Honeycrisp’ apple. Retracting nets before harvest improved red coloration without increasing sunburn risk.	High installation cost; effectiveness depends on net type, shading level, cultivar, and possible reduction in red color development.	[50]
Evaporative cooling and overhead irrigation	Removes fruit heat through water evaporation and directly reduces fruit-surface temperature during extreme heat events	Pulsed overtree irrigation reduced visible solar injury by 15.8% (1991) and 9.4% (1992). Maximum fruit-surface temperature was reduced by 8.1 °C when untreated fruit reached 45.6 °C.	Requires sufficient water availability and irrigation infrastructure; excessive cooling may influence soluble solids and acidity.	[62]
Reflective groundcovers and orchard mulches	Reflects and redistributes solar radiation within the canopy, improving light distribution while reducing excessive direct radiation exposure	Shade netting reduced sunburn incidence by 76–80% in ‘Gala Baigent’ and ‘Fuji Raku Raku’ apples. Reflective mulch increased premium-colored fruit by 27% and 9% in ‘Gala Baigent’ and ‘Fuji Raku Raku’, respectively.	Installation and maintenance costs vary according to orchard size; excessive reflected radiation may increase fruit heat load if not combined with other protection methods.	[47]
Reflective particle films (processed kaolin coatings)	Forms a reflective coating on fruit surfaces, reducing absorption of UV and solar radiation and lowering fruit temperature	Application of processed-kaolin particle films at 45–56 kg ha^−1^ and 3–12% concentrations effectively suppressed solar injury. Fruit temperature reduction was proportional to particle residue coverage.	Requires repeated applications and uniform fruit coverage; effectiveness depends on persistence, rainfall, and application timing.	[63]
Canopy management and orchard architecture optimization	Regulates light interception, fruit exposure, and canopy microclimate through training systems, pruning, and fruit positioning	Fruit position strongly influenced light exposure and quality; top-canopy fruits received approximately 43% available light, while inner-canopy fruits received about 12%. Excessive exposure increased sunburn risk.	Incorrect pruning or excessive leaf removal may expose previously shaded fruit and increase photooxidative sunburn; requires skilled management.	[43]
Biostimulants and antioxidant treatments	Enhances antioxidant defense systems, improves stress tolerance, and reduces oxidative damage caused by heat and radiation stress	Preharvest biostimulant applications improved physiological responses, reduced sunburn severity, and maintained fruit quality in ‘Gala’ apple orchards.	Responses depend on formulation, application timing, cultivar, and environmental conditions; repeated applications increase production costs.	[5]
Stress-protective foliar regulators (Ca/Si-based treatments and PGRs)	Strengthens fruit-surface stability, improves stress resistance, and supports cellular protection under heat stress	Calcium carbonate-based foliar sprays showed potential to protect fruit quality during extreme heat events by reducing thermal stress effects.	Field effectiveness varies with formulation, stress intensity, and application timing; economic feasibility requires orchard-scale evaluation.	[7]
Irrigation and water status management	Maintains plant water status and reduces thermal stress by improving transpiration-based cooling capacity	Water stress increased sunburn susceptibility, whereas maintaining adequate irrigation reduced heat-related fruit damage and improved fruit resilience.	Water availability is a major limiting factor, particularly in arid and semi-arid production regions.	[64]
Cultivar selection and genetic resistance approaches	Utilizes genetic variation in peel pigments, cuticle properties, antioxidant capacity, and heat-response traits for developing tolerant cultivars	Sunburn severity in ‘Buckeye Gala’ was associated with reduced anthocyanin content, altered cuticle characteristics, chloroplast degradation, and changes in phenolic compounds. DA meter analysis showed potential for rapid prediction of sunburn severity.	Breeding requires long-term evaluation and multi-environment testing because tolerance is strongly influenced by genotype × environment interactions.	[46]
Integrated sunburn management (ISM)	Combines complementary approaches including netting, evaporative cooling, reflective materials, and orchard management to optimize heat and radiation control	Evaporative cooling can reduce fruit-surface temperature by up to 8.5 °C, while protective netting can maintain fruit temperatures approximately 5–10 °C lower than un-netted fruit under similar conditions.	Requires higher initial investment and decision-making based on cultivar, climate risk, water availability, and economic conditions.	[7]

## Data Availability

No new data were created or analyzed in this study. All information supporting this study is included in the article.
